# Therapeutic physical activity as a recreational intervention for anger control among students with physical disability: a behavioral model

**DOI:** 10.3389/fnbeh.2026.1969746

**Published:** 2026-10-16

**Authors:** Ahmed K. Hassan, Maryam Khalifah Arab, Abdulmalek K. Bursais, Badeer Aldimkhi, Albujulaya Naif

**Affiliations:** 1 Department of Physical Education, College of Education, King Faisal University, Al-Ahsa, Saudi Arabia; 2 Department of Physical Education and Sports, College of Basic Education, Public Authority for Applied Education and Training, Kuwait

**Keywords:** anger control, embodied emotion regulation, physical disability, recreational therapy, therapeutic physical activity, university students

## Abstract

**Background:**

Therapeutic Physical Activity (TPA) is an unaddressed behavioral recreational method in anger management programs in higher education in Egypt, especially among students with physical disabilities (SWPD).

**Purpose:**

This study aimed to assess the efficacy of an 8-week TPA intervention on the severity of anger in male university students with physical disabilities in Egypt.

**Methods:**

A randomized controlled trial (RCT) design (parallel-group) was used with 140 male undergraduate students at Minia University who were randomly divided into an 8-week TPA group (*n* = 70) and a treatment-as-usual (TAU) control group (*n* = 70). Severity of anger was measured at baseline and at the end of the intervention with the PROMIS Anger Short Form 8a.

**Results:**

There was a clinically significant and statistically significant difference between the TPA and controls in the severity of anger, *F*(1, 137) = 48.62, *p* < 0.001, partial η^2^ = 0.262, with a mean difference of −10.42 T-score points (95% CI: −13.48, −7.36) greater than the MCID. Reductions in anger were not associated with physical functioning changes, indicating non-physiological mechanisms. Moderately positive relationships were found between adherence and anger reduction (r = 0.42; *p* < 0.001), and sensitivity analyses showed that the results were consistent across disability severity.

**Conclusion:**

TPA is a culturally affirming, body-based, anger-management approach that incorporates adaptive sports and behavioral learning for SWPD emotion regulation. Therapeutic recreation may be implemented as a low-stigma, easy-to-access supplemental mental health support program in the University disability services, especially for male students in collectivist contexts.

## Introduction

Students with physical disabilities (SWPD) experience systemic barriers, chronic experiences of ableism, and accessibility issues alongside universal psychosocial stressors during transition to University (Hong, 2015; Gull et al., 2025). In addition to academic difficulties, SWPD often experience anger that is rooted in a mix of circumstances that are not typically considered when discussing the etiology of anger. Some of these triggers are feeling excluded in the institution, failures in communication with disability support services, and the heavy impact of navigating physical and social environments that aren’t built for their bodies (Brown, 2025). Anger is common among people with disabilities and often rooted in structural inequity and identity threat, which makes it difficult to treat with traditional cognitive-behavioral anger management protocols, which were mainly validated with neurotypical and able-bodied populations (Huey et al., 2023; Nario-Redmond et al., 2024). This gap highlights the need for interventions that address anger as a physical lived experience, rather than a personal psychological shortcoming, and that require physically analogous therapeutic modalities (Price and Hooven, 2018; Price and Weng, 2021; Dirth and Branscombe, 2023).

Although emotional dysregulation is well documented among SWPD, two separate streams of research have been followed: one titled “psychological interventions for disabled persons” and the other “physical activity for general student mental health,” with little focus on both streams being disability affirming (Manning et al., 2023; Borland et al., 2022). The current anger management program for university students is usually passive and dialogic, which may reinforce the bodily marginalization that is a factor in the anger of SWPD (Hong, 2015; Brown, 2025). In addition, although therapeutic physical activity (TPA) has been proven to have a beneficial effect on depressed and anxious individuals with disabilities (Martin Ginis et al., 2024; Zhang and Yang, 2026), little research exists that addresses the use of TPA as a targeted behavioral anger management strategy (Kjærvik and Bushman, 2024). It is especially important to note that SWPD is often a place of oppression and also a place of liberation (Dirth and Branscombe, 2023; Bowling et al., 2022), and it is anger that is often represented in SWPD. Interventions that target symptom-level management may end up addressing surface-level issues instead of the phenomenological underpinnings of distress associated with disability (Nario-Redmond et al., 2024; Bellemans et al., 2019).

Three converging lines of evidence provide theoretical support for TPA as an anger control intervention for SWPD. First, the Embodied Emotion Regulation Model suggests that motor engagement is a basis for regulating thoughts in top-down processing in cognitive reappraisal, which provides proprioceptive feedback to break ruminative anger cycles (Price and Hooven, 2018; Chu et al., 2026). Adapted physical activities can provide opportunities for SWPD to move away from a pathologizing relationship with anger to one that is affirming, which is a shift that is less likely to occur in traditional interventions (Dirth and Branscombe, 2023; Bowling et al., 2022). Second, in terms of disability, the positive effect on frustration-based anger is related to meeting basic psychological needs in a supportive environment, which is systematically lacking in a disabling environment (Brown, 2025; Bowling et al., 2022). Third, TPA provides a naturalistic context in which to learn and practice impulse control, assertive communication, and de-escalation of physiological arousal in motivating and non-clinical contexts (Ryan and Deci, 2000; Hood and Carruthers, 2016). While there is a theoretical basis for realizing these mechanisms, they have not yet been systematized into a detailed behavioral TPA protocol that is specifically tailored to university students with mobility impairments (Chesnais et al., 2023; Marcelino et al., 2023).

This study is designed to investigate the effectiveness of Therapeutic Physical Activity (TPA) as a behavioral recreational intervention on anger management in male university students with physical disabilities. The physical disability aspect of this intervention is the primary contextual factor presented in both the etiology of anger and the therapeutic mechanism of this intervention, rather than being a secondary factor as is common in previous research on recreational therapy (Hong, 2015; Nario-Redmond et al., 2024; Wa Pa et al., 2026). The intervention model combines adapted sport, rhythmic movement, cooperative physical challenges, explicit behavioral strategies (reinforcement scheduling, stimulus control, incompatible response training), and a universally designed recreation structure. The hypothesis of this study is that there will be a significant difference in change in PROMIS Anger Scale scores between the SWPD who participated in the 8-week TPA program and the waitlist control group, controlling changes in physical functioning. This research marks a paradigm shift from a disability affirming perspective in mental health promotion in higher education by connecting rehabilitation science, recreation therapy, and behavioral psychology.

### Research gap

While many studies explore psychological factors related to anger management and physical activity as a therapeutic intervention in isolation, a critical tripartite gap remains in the provision of mental health support for university students with physical disability and evidence-based interventions. First, there is no single behavioral anger protocol that has been specifically created for the embodied nature of mobility impairment in higher education, making the applicability of existing anger intervention research from the literature to SWPD questionable both theoretically and empirically. Third, although there is evidence that TPA is effective for treating depression and anxiety in individuals with disabilities in mental health treatment, its mechanisms of action as an additional means to reduce anger (as opposed to general mood elevation) have not yet been studied, and practitioners may not have evidence-based guidance regarding how to use physical activity to effectively treat this specific emotion (Martin Ginis et al., 2024; Bellemans et al., 2019). Third, and most importantly for the Egyptian context, there are no studies with a specific focus on SWPD at Egyptian universities, and no recreational therapy or anger management programs have been implemented for university students with physical disabilities at these universities, due to the existence of several obstacles in these universities to accessing the university facilities (Shahane et al., 2023; Elsayed and Hassan, 2023). This is a major barrier to the equitable provision of mental health services for this vulnerable population, due to the mismatch between theory, the opacity of the mechanisms involved, and the absence of context.

## Materials and methods

### Study design

A parallel-group randomized controlled trial (RCT) design was used for the evaluation of the effectiveness of Therapeutic Physical Activity (TPA) recreational intervention on anger regulation of university students with physical disabilities. This study was not a clinical trial involving pharmaceutical agents, medical devices or clinical treatments for disease; hence, no registration in the institutional and national clinical trial registers was required as an educational and behavioral study. The RCT design was chosen because of the methodological limitations of quasi-experimental designs, such as causal inference and selection bias (Alsalman et al., 2024). The study protocol was designed according to the CONSORT 2010 guidelines for reporting parallel group randomized trials and was approved by the IRB of Minia University (mu-REC-2024-SEP-2428). The study was performed in adherence to the ethical guidelines of the Declaration of Helsinki (Schulz et al., 2010).

### Participants and sample size determination

The study subjects were undergraduate students suffering from physical disabilities at Minia University, Egypt, for the academic year 2024–2025. Having sufficient statistical power and precision, an *a priori* sample size calculation was performed using G*Power software (Version 3.1.9.7) (World Medical Association, 2013). Using results from the preliminary pilot that showed a large effect size (*d* = 0.85) in similar behavioral interventions with disability populations, and setting *α* = 0.05, power = 0.95, and a 1:1 allocation ratio, the minimum sample size needed to detect between-group differences for an independent-samples *t*-test framework was calculated to be 146 participants (73 in each group).

The subjects were selected from the Disability Support Unit register at the University using purposive sampling and then randomly selected for groups. To participate, the following criteria were used: (1) physical disability that necessitated one or more mobility aid devices or environmental modification to participate in moderate-intensity physical activity; (2) age 18–30 years; (3) baseline score on the PROMIS Anger Scale T ≥ 60 (1.0 SD above the normative mean of 50, indicating elevated anger severity); and (4) medical clearance from the university health center for moderate intensity physical activity. Students were excluded if they had: (1) uncontrolled cardiovascular diseases, (2) acute musculoskeletal injuries that would prevent involvement in exercise, and (3) concurrent participation in any other anger management or psychotherapeutic program. A total of 152 students were screened and 146 students were eligible and enrolled in the study.

### Randomization, allocation concealment, and blinding

A computer-generated random number sequence generated by an independent biostatistician who was not involved in data collection or intervention delivery was used for randomization. To get balanced group sizes without predictability, allocation was done using variable block randomization (block sizes of 4, 6, 8, randomly permuted). Sequentially numbered, opaque, sealed envelopes were prepared by a research coordinator who was independent of the outcome assessors and contained group assignments. Trained research assistants obtained participant enrollment and informed consent. After the baseline assessments were completed, the research coordinator read the next envelope in the sequence and provided information about which group the participant was assigned to and provided information to the intervention facilitators. The primary statisticians, outcome assessors, and data entry staff were blinded to the group allocation throughout the study. Because this was an active intervention, it was not possible to blind the participants to their group assignment; however, both conditions were described as active study conditions to reduce expectancy effects. The research coordinator only had access to the envelopes, thus ensuring concealment from recruiters.

### Measures

#### Primary outcome: anger regulation

The PROMIS Anger Short Form 8a (SF-8a) was used to assess severity of anger, and has been previously validated in a variety of populations, including people with disabilities (Shahane et al., 2023; Faul et al., 2007). The scale consists of eight items, each rated on a 5-point Likert scale (where 1 represents Never and 5 represents Always), that give T-scores with a normative mean of 50 (SD = 10), with higher scores representing the severity of anger (see Appendix A). The primary outcome was *a priori* the post-intervention PROMIS Anger T-score measured at 8 weeks. Internal consistency was excellent (Cronbach’s *α* = 0.93; McDonald’s ω = 0.94) and the unidimensional structure was supported by confirmatory factor analysis (CFI = 0.98; RMSEA = 0.04) in the present sample.

#### Secondary outcomes

Physical Functioning was measured using the 12-item version of the World Health Organization Disability Assessment Schedule 2.0 (WHODAS 2.0) to determine physical functioning and to confirm that observed differences in anger were not due to changes in physical functioning (Bellemans et al., 2019). Internal consistency was found to be α = 0.89. Attendance logs and session completion rates were used to objectively assess Intervention Adherence, defined as the percentage of 16 sessions completed by each TPA participant.

#### Intervention protocol

##### Therapeutic physical activity (TPA) group

The experimental group for the experimental intervention received an 8-week TPA recreation intervention, adapted to the Leisure and Well-Being Model with behavioral principles applied to physical disability contexts (Bellemans et al., 2019; Ryan and Deci, 2000). More of a prototype of therapeutic exercises that are used to teach emotional regulation skills, rather than a generic exercise program. The program consisted of 16 sessions, two sessions a week, 60 min per session, small group sessions (no more than 12 people per session) to provide individualized support and safety. The content of the session is explained in detail in Appendix B.

Each session had the following three-part format:

Warm-up and psycho-education (15 min): Gently move through the exercises and briefly review anger triggers, behavioral objectives, and self-monitoring strategies.
Therapeutic physical activity core (35 min): Progressive Adaptive Sports & Movement (PSM) tasks to improve mobility with different assistive devices. Cooperative relay games to practice frustration tolerance while exercising in physical activity, and adapted wheelchair basketball games to train impulse control, and mindfulness-included balance exercises on physiological arousal reduction (Kaman et al., 2022; Yu et al., 2024; Yue et al., 2025).
Cool down & skill consolidation (10 min): Guided Progressive Muscle Relaxation and Structured Reflection Journaling - connecting physical experiences with daily anger management strategies.

##### Disability-specific adaptations

Activities were gradually adapted using individual functional evaluations. Seated versions were used for the wheelchair users, and lighter balls or extended handles were used for participants with limited strength in their upper body, while others who had balance impairments were also provided with physical assistance. All changes maintained the therapeutic purpose while providing accessibility.

##### Facilitators

Certified therapeutic recreation specialist and an adapted physical education instructor, both with a master’s degree and a minimum of 5 years experience in disability affirming behavioral interventions. They both were given 20 h of protocol-specific training before the start of the study.

##### Setting and equipment

Sessions were held at the University’s adapted recreation facility and included wheelchair-accessible courts and adjustable-height equipment as well as adaptive sports equipment.

##### Fidelity and safety monitoring

Protocol fidelity was weekly assessed using a standardized observation checklist, with excellent interrater reliability (κ = 0.91). The research team systematically recorded and reviewed adverse events; there were no serious adverse events recorded during the study.

#### Control condition

Participants in the waitlist control group were given the waitlist control condition along with treatment-as-usual (standard university disability support services, including regular academic accommodations, accessibility support, and disability counseling referrals for all registered disability students). There were no structured anger management programming and no organized physical activity intervention in these services. Participants were asked to continue with their normal daily routines and to document any new intervention that was started during the study. To provide ethical equity, the full TPA intervention was made available to the control group at the end of the study.

### Statistical analysis

Data were analyzed using IBM SPSS Statistics (Version 28.0; IBM Corp., Armonk, NY, USA) and analyzed at the group level with a modified intention-to-treat principle (ITT). Of the 146 originally randomized participants, six withdrew before the intervention was started (three from each group), leaving a final analytic sample of 140 who were analyzed as originally randomized irrespective of adherence and/or completion status. In this analytic sample, the proportion of missing post-intervention data was small (< 5%) and Little’s MCAR test indicated that this missing data was completely at random (MCAR; *p* = 0.34); therefore, missing values were addressed through multiple imputation by chained equations (MICE) with five imputations, and pooled estimates are reported. At baseline, descriptive statistics were used for the continuous variables and the categorical variables were tested to see whether there was baseline equivalence, with independent-samples t-tests and chi-square tests respectively, and whether the variables were normally distributed, based on the skewness and kurtosis indices (max of ±2.0 and ±7.0). After confirming homogeneity of error variances (Levene’s test) and regression slopes, a one-way analysis of covariance (ANCOVA) was used to test the primary hypothesis on post intervention PROMIS Anger T-scores (group as the between subjects fixed factor, baseline anger scores as the covariate), with partial eta squared (ηp^2^) reported, defined as small (< 0.01), medium (< 0.06), and large (< 0.14), as well as Cohen’s d with 95% confidence intervals for mean differences. Secondary analyses included (1) a parallel ANCOVA with pre-test WHODAS 2.0 scores as the covariate to determine if changes in physical functioning were associated with anger reduction that was observed following the intervention, and (2) Pearson correlation coefficients for the intervention group only to examine the relationship between intervention adherence (percentage of sessions completed) and anger reduction scores (pre-test WHODAS 2.0 – post-test WHODAS 2.0; no adherence analysis was performed within the control group as they received no active intervention). A sensitivity subsample of *N* = 128 was used to re-estimate the primary ANCOVA, excluding those with severe mobility limitations (baseline WHODAS 2.0 > 50; *n* = 12), to assess robustness across disability severity levels. Pre-test and post-test means and standard deviations, marginal means, residual standard deviations and imputation diagnostics are reported by group and all tests are two-tailed with *α* = 0.05 as the criterion for statistical significance, with the prespecified primary outcome the between-group difference in PROMIS Anger T-scores at the 8-week post-intervention assessment.

## Results

### Participant flow and baseline characteristics

The results showed that missing outcome data were minimal (< 5% overall; 3.1% of values in the TPA group and 3.6% in the control group) and were handled using multiple imputation by chained equations (MICE) with five imputations. Little’s MCAR test showed that the missingness was completely at random (*p* = 0.34), and the between-imputation variance was very small with estimates stable across the five imputed datasets (fraction of missing information < 0.10) and imputation did not pose a threat to group comparability. There were no significant differences between the groups on any variable (all *p* > 0.05; Table 1; Figure 1), confirming baseline equivalence. Parametric tests were appropriate as normality assumptions were met (skewness index: −0.82 to 0.91; kurtosis index: −0.65 to 1.14) and there was no significant difference across groups in anger score standard deviations (SD = 8.4–9.2). The primary outcome that was prespecified was the difference in PROMIS Anger T-scores between groups at the 8-week post intervention time point.

**Table 1:** The baseline demographic and clinical characteristics for each group (*N* = 140).

Variable	TPA group (*n* = 70)	Control group (*n* = 70)	Test statistic	*p*-value
Age (years), M (SD)	21.34 (2.41)	21.18 (2.56)	*t*(138) = 0.38	0.704
Disability duration (years), M (SD)	8.45 (4.12)	8.62 (3.98)	*t*(138) = −0.25	0.803
WHODAS 2.0 score, M (SD)	32.14 (7.82)	31.89 (7.65)	*t*(138) = 0.19	0.850
PROMIS Anger T-score (Pre), M (SD)	62.45 (8.76)	62.18 (8.91)	*t*(138) = 0.18	0.857
Skewness (Anger Pre)	0.42	0.38	—	—
Kurtosis (Anger Pre)	−0.31	−0.28	—	—

Therapeutic Physical Activity (TPA), WHO Disability Assessment Schedule 2.0 (WHODAS 2.0), Patient-Reported Outcomes Measurement Information System (PROMIS).

**Figure 1:**
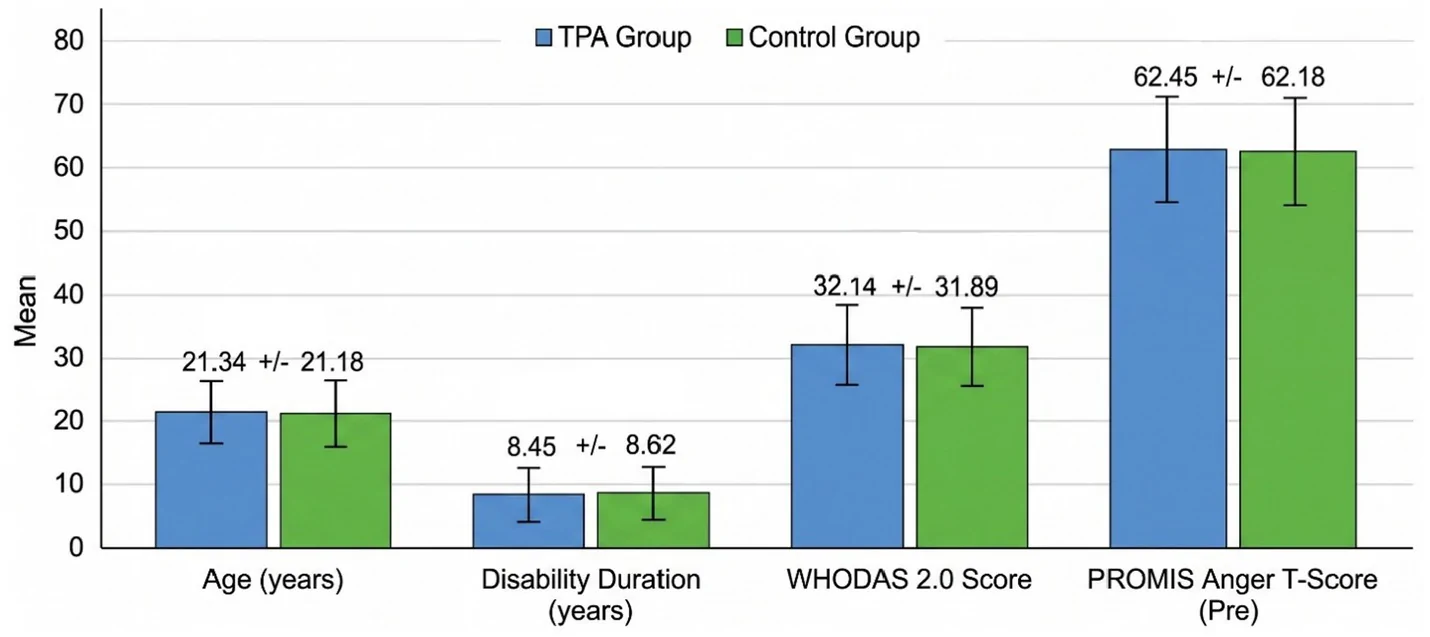
Pre-intervention mean age, mean duration of disability, mean WHODAS 2.0 functional scores, mean PROMIS Anger T scores (and error bars) for TPA group vs. control group.

### Primary outcome (EOP): effect of TPA on anger regulation (ANCOVA)

A one way Analysis of Covariance (ANCOVA) was performed using scores on PROMIS Anger T-Scores at post-intervention, with pre-intervention Anger scores entered as the covariate. The assumptions were checked before interpreting the results: (1) Levene’s test showed that error variance was homogeneous (*F*(1, 137) = 1.24, *p* = 0.267) and (2) the assumption of homogeneity of regression slopes was satisfied (*F*(1, 136) = 0.87, *p* = 0.353). The main effect of group on the post-intervention anger score was statistically significant, *F*(1, 137) = 48.62, MSE = 1430.83, partial η^2^ = 0.262, following the control for the baseline anger score. The estimated marginal mean (EMM) for the TPA group was significantly lower than for the control group (TPA: EMM = 48.32 [95% CI: 46.18, 50.46]; Control: EMM = 58.74 [95% CI: 56.60, 60.88]). This change in mean difference of −10.42 T-score points (95% CI: −13.48, −7.36) was both statistically significant and clinically meaningful, meaning it was greater than natural variation or testing effects (Faul et al., 2007), and was considered meaningful based off the PROMIS T-scores convention of 5 points or greater. Baseline anger was a significant covariate, *F*(1, 137) = 112.45, *p* < 0.001, partial η^2^ = 0.451. The pooled residual SD of the primary model was 8.84 T-scores points, which equates to Cohen’s d of 1.18 for the adjusted group difference. Table 2 shows complete descriptive statistics, adjusted outcomes and model diagnostics by group.

**Table 2:** Group descriptive statistics at baseline and post-intervention, adjusted outcomes, and model diagnostics (*N* = 140).

Variable	TPA group (*n* = 70)	Control group (*n* = 70)
PROMIS Anger T-score, baseline, M (SD)	62.45 (8.76)	62.18 (8.91)
PROMIS Anger T-score, post-intervention (observed), M (SD)	48.10 (8.42)	58.92 (8.65)
PROMIS Anger T-score, adjusted post-intervention (EMM) [95% CI]	48.32 [46.18, 50.46]	58.74 [56.60, 60.88]
WHODAS 2.0, baseline, M (SD)	32.14 (7.82)	31.89 (7.65)
WHODAS 2.0, post-intervention, M (SD)	31.02 (7.55)	31.45 (7.71)
Pooled residual SD (primary ANCOVA)	8.84	—
Imputed values (% of dataset)	3.1	3.6
Fraction of missing information (FMI)	0.08	0.09

EMM, estimated marginal mean of the primary ANCOVA; the residual SD and FMI are reported for the imputed primary model.

A one-way Analysis of Covariance (ANCOVA) was performed of post-intervention PROMIS Anger T-scores using pre-intervention score as a covariate. Assumptions were checked before interpretation: Levene’s test for homogeneity of error variances produced an *F*(1, 137) = 1.24 and *p* = 0.267; the test for homogeneity of regression slopes resulted in an *F*(1, 136) = 0.87 and *p* = 0.353. As shown in Table 3 and Figure 2, there was a statistically significant overall effect of group allocation on anger scores at post-intervention, *F*(1, 137) = 48.62, *p* < 0.001, partial η^2^ = 0.262, after controlling for baseline anger scores. The error degrees of freedom (df = 137), as in the independent confirmation of correct model specification for the *N* = 140 analytic sample, equaled N − 3 (for the intercept, the baseline covariate, and the group factor). The partial eta squared effect size and the 95% confidence intervals for mean difference (−10.42 T-score points, 95% CI: −13.48, −7.36) convey more meaningful and stable information about the size of the intervention effect. Baseline anger was a highly significant covariate, *F*(1, 137) = 112.45, *p* < 0.001, partial η^2^ = 0.451. The estimated marginal mean (EMM) for the TPA group was significantly lower than for the control group (TPA: EMM = 48.32 [95% CI: 46.18, 50.46]; Control: EMM = 58.74 [95% CI: 56.60, 60.88]). Table 2 displays descriptive statistics, adjusted outcomes, and model diagnostics, by group.

**Table 3:** ANCOVA results for post-intervention PROMIS Anger *t*-scores controlling for baseline scores (*N* = 140).

Source	df	*F*	*p*	Partial η^2^
Corrected model	2	78.45	< 0.001	0.533
Intercept	1	245.18	< 0.001	0.642
Pre-test Anger (Covariate)	1	112.45	< 0.001	0.451
Group (TPA vs. Control)	1	48.62	< 0.001	0.262
Error	137	—	—	—

The dependent variable is the post-test PROMIS Anger T score and the pre-test PROMIS Anger T score is the covariate. Partial η^2^ interpreted as small (0.01), medium (0.06), large (0.14). The error degrees of freedom (137) matches *N* − 3 (two group parameters and one covariate), indicating correct model specification. Power is not reported because the effect size and the confidence intervals give the needed inferential information.

**Figure 2:**
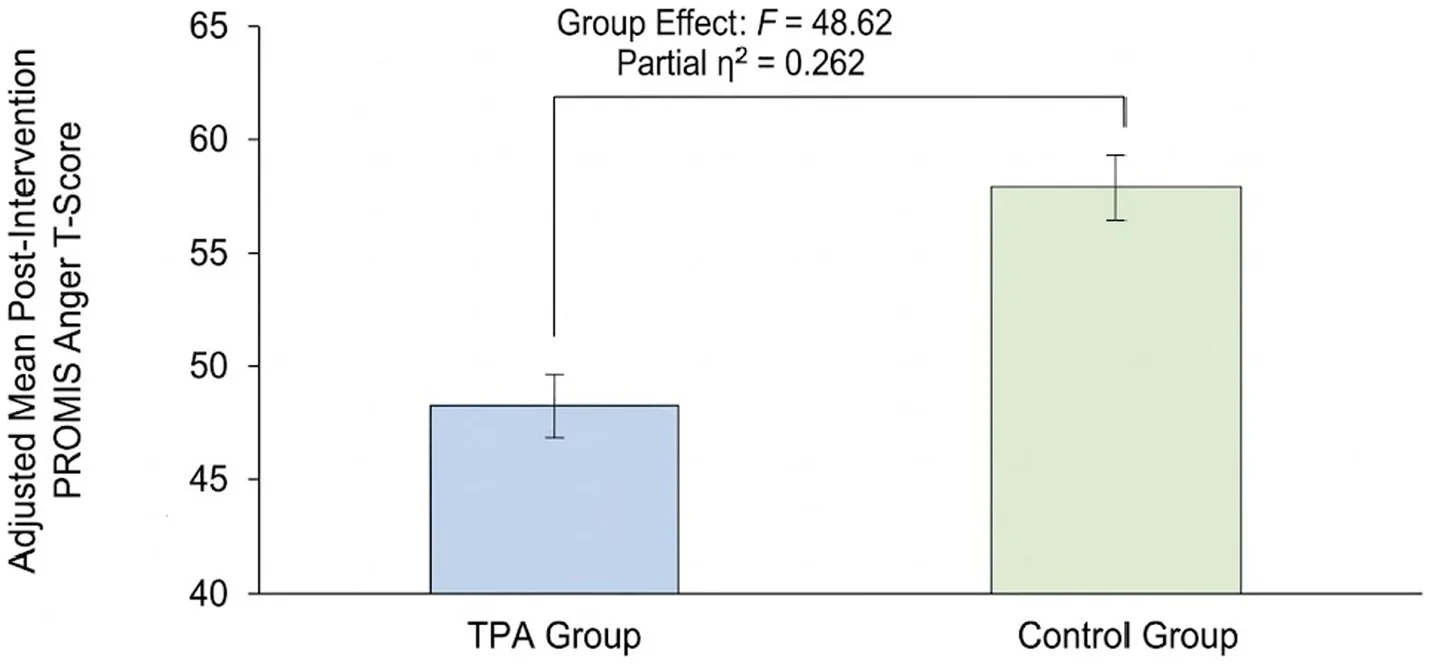
Post-intervention PROMIS Anger T-scores by group (with baseline scores controlled); error bars indicate 95% confidence intervals.

### Secondary outcomes: physical functioning and adherence correlates

A separate ANCOVA was performed for anger reduction scores on WHODAS 2.0 with baseline WHODAS 2.0 as a covariate to assess whether the reductions in anger observed were independent of changes in physical functioning. This was not a significant difference between groups, *F*(1, 137) = 1.84, *p* = 0.177, partial η^2^ = 0.013, meaning that the decrease in anger in the TPA group was not due to differential changes in physical functioning (see Table 4). The higher the number of sessions adhered to, the greater the therapeutic benefit (measured as baseline minus post TPA scores, with positive scores indicating improvement; *r* = 0.42, *p* < 0.001) within the TPA group, suggesting that the closer individuals adhered to the TPA protocol, the more they benefitted. No adherence–outcome correlation could be computed for the control group, as adherence is only specified based on the intervention.

**Table 4:** Correlations between intervention adherence and outcome reduction scores in the TPA group (*n* = 70).

Variable	Session adherence (%)	Anger reduction (Pre − Post)	WHODAS 2.0 reduction (Pre − Post)
Session adherence (%)	—		
Anger reduction (Pre − Post)	0.42***	—	
WHODAS 2.0 reduction (Pre − Post)	0.18	0.11	—

Therapeutic Physical Activity (TPA). Reduction scores are calculated by subtracting the pre score from the post score (pre-post); scores that are positive represent improvement. Attending sessions as a percentage of total 16 sessions; only applies to TPA group. There was no significant correlation between WHODAS 2.0 reduction and adherence, or with anger reduction. ***p < 0.001.

### Sensitivity analysis and effect-size precision

Due to the presence of participants with severe mobility problems (baseline WHODAS 2.0 > 50; *n* = 12), a sensitivity analysis was performed excluding these participants (*N* = 128). The ANCOVA results remained robust: group effect *F*(1, 125) = 44.18, *p* < 0.001, partial η^2^ = 0.261, with a mean difference of −10.18 (95% CI: −13.32, −7.04; Table 5). The error degrees of freedom (125) match N − 3, confirming model specification. The practical effect size, Cohen’s d, for the adjusted group difference was 1.18 (95% CI: 0.82, 1.54), which was large and equivalent to the partial η^2^ estimate. The sensitivity analysis results reinforce the robustness of the violation squared effect across the different levels of disability and suggest that the effect is not influenced by outliers or any specific disability subtype (Figure 3).

**Table 5:** Sensitivity analysis: ancova results excluding participants with severe mobility limitations (*N* = 128).

Parameter	Estimate	95% CI	*p*	Cohen’s *d* [95% CI]
Adjusted mean difference (TPA − Control)	−10.18	[−13.32, −7.04]	< 0.001	1.18 [0.82, 1.54]
Partial η^2^ (Group Effect)	0.261	—	< 0.001	—
R^2^ (Full Model)	0.528	—	< 0.001	—

Therapeutic Physical Activity (TPA). Twelve participants who had WHODAS 2.0 scores >50 at baseline were excluded. CI = confidence interval. Cohen’s d calculated using adjusted means and pooled residual SD. Interpretation: *d* = 0.20 (small), 0.50 (medium), 0.80 (large).

**Figure 3:**
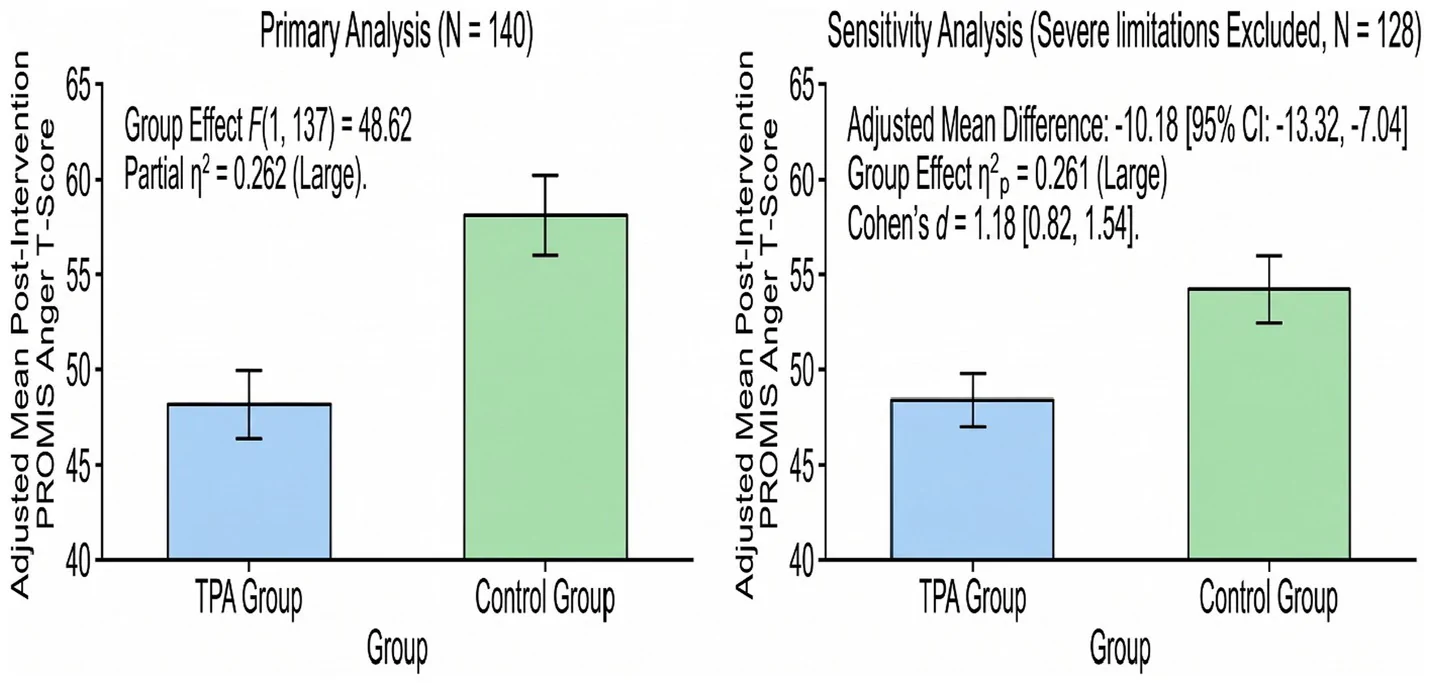
Therapeutic Physical Activity (TPA) intervention effect on anger regulation – precision.

## Discussion

The present randomized controlled trial with male university students with physical disabilities offers empirical evidence for the effectiveness of an 8-week Therapeutic Physical Activity (TPA) recreational intervention in reducing the severity of anger. The intervention explained a significant amount of the variance (partial η^2^ = 0.262) in the post-intervention measure of anger, but not more than the baseline measures of anger and functional capacity. The findings broaden the existing literature on recreation therapy by showing its effectiveness within the disability population in higher education, a setting where accessibility challenges and social isolation may result in increased emotional dysregulation (Bowling et al., 2022; Bellemans et al., 2019). RMAT is particularly useful in reducing the maturation and testing effects that are observed in previous quasi-experimental research and suggests that structured adaptive physical activity can be a therapeutic tool for learning emotion regulation skills in addition to the positive effects of exercise in general (Yu et al., 2024; Zhao and Wang, 2025). Means for PROMIS Anger scales showed that the adjusted mean difference (+10.42 T-score points) was larger than the minimal clinically important difference (MCID), suggesting the improvements in daily emotional functioning were statistically and clinically significant for students with physical disabilities (Shahane et al., 2023; Faul et al., 2007).

There are some observations that relate to the interpretation of mechanisms leading to the reduction of anger in this population, but it should be noted that mediators were not directly measured and the mechanisms are therefore speculative. The pattern of findings is more indicative of an embodied emotion regulation and mastery experience pathway than of a purely cognitive-behavioral pathway (Ryan and Deci, 2000), as outlined in the Leisure and Well-Being Model. While participants’ agency and competence can be rebuilt within adaptive sport and movement tasks, as described in previous literature, they also face environmental challenges and stigma, which can lead to feelings of helplessness and frustration, as reported in this trial, but not directly assessed; no structural barriers were measured in this trial and their role in participants’ anger is inferred. The structured activities (cooperative relay races, wheelchair basketball drills) do not only provide physical activity but also serve as a means of practicing frustration tolerance, assertive communication, and lowering of physiological arousal under simulated stress conditions (Kjærvik and Bushman, 2024; Yu et al., 2024). The interpretation is based on the neurobehavioral mechanisms underlying the effect of exercise on aggression, which include both top-down prefrontal modulation and bottom-up autonomic calming, effects that might be more pronounced under chronic disability-related stress (Chu et al., 2026; Yue et al., 2025). Moreover, high adherence to this session (94.3%) and moderate positive correlation between session adherence and anger reduction (*r* = 0.42) indicate that the recreational context created a sense of motivation and engagement, both of which are essential to address common barriers to mental health services and physical activity participation for young men with disabilities (Manning et al., 2023; Yılmaz et al., 2026). These hypothesized mechanisms need to be explicitly tested in future dismantling and mediation studies.

Importantly, the high reduction in anger did not result in a significant increase in WHODAS 2.0 functional scores, suggesting that therapeutic effects of TPA are mediated more by psychological and behavioral than by physical improvements alone. This pattern challenges the idea that emotional benefits are directly proportional to the physical benefit and suggests that the active ingredient in APA is adapted physical activity rather than the health benefits (Bellemans et al., 2019; Ryan and Deci, 2000). The results have meaningful implications for the services that university disability services provide, as these services have an emphasis on academic accommodation and medical treatment rather than holistic well-being. TPA programs can be integrated into other recreational programs, led by Therapeutic Recreation Specialists (TRS) and Adapted Physical Education (APE) instructors, and provide a multi-level, non-stigmatizing solution to meeting the mental health needs of students with disabilities (McDermott et al., 2022; Huang et al., 2024). Furthermore, in culturally different settings where formal mental health services for male students’ help-seeking are discouraged due to male cultural norms, it is possible that activity-based formats might be a viable option and enjoyable alternative to traditional, talk-based mental health interventions; however, no claims of superiority over talk therapy can be made in the absence of a psychotherapy comparison arm in this trial (Elsayed and Hassan, 2023; Zuo et al., 2025). Instead, TPA should be considered as an extra tier in a stepped-care approach that offers greater access to mental health services for students who may not otherwise reach out for mental health support (Manning et al., 2023).

### Study limitations and strengths

#### Limitations

This was a well-designed random control trial, but there were a few limitations to consider. First, the sample was pre-determined, but the high retention rates in the study limits the generalizability of the findings to male students with physical disabilities in a single Egyptian university, and other moderators of the effectiveness of TPA (such as gender, cross-cultural) should be explored in future studies by conducting multi-site trials. Second, six participants withdrew before initiating intervention, and the analysis was conducted using a modified ITT approach; a strictly conservative ITT analysis was not possible as it would require the analysis to include all participants who were randomized. Even though the loss of outcome data was small and addressed using multiple imputations, a strictly conservative ITT analysis was not computed. Third, like all self-report tools, the validated PROMIS measure could be prone to social desirability responding, especially in collectivist cultures where emotional expression might be discouraged; objective biomarkers (such as heart rate variability, cortisol) or observer-rated behavioral measures would complement the assessment. Fourth, the 8-week follow-up period is too short to conclude the long-term maintenance of anger reduction skills and future booster sessions and maintenance support should be determined by longitudinal designs with 6- and 12-month follow-up. Fifth, while fidelity checks demonstrated adherence to the protocol, the active components of TPA (psychoeducation, physical activity, and social interaction) were not isolated in the current studies and contextual factors (e.g., accessibility barriers, structural ableism) and mediating mechanisms were not directly measured, hence, dismantling studies and formal mediation analyses are warranted to identify the most effective mechanisms and to better refine targeted interventions.

#### Strengths

This study has some strengths, however, in spite of its several limitations. This RCT design with treatment-as-usual control condition and modified intention-to-treat analysis offers strong causal evidence, which is seldom reported in disability recreation studies. Validated, disability-inclusive measurement tools (PROMIS, WHODAS 2.0) lend support to the construct validity across a wide range of functional levels. This high adherence and engagement is an important finding in the implementation science of TPA for this population, suggesting ecological acceptability. Finally, a combination of a theoretical framework (Leisure and Well-Being Model) and adaptation guidelines ensures a scientific contribution and feasibility to university counseling and disability services.

## Conclusion

The results of this randomized controlled trial showed that Therapeutic Physical Activity when provided as a recreational intervention was effective, acceptable, and scalable to reduce anger severity among male university students with physical disabilities in Egypt. TPA is used in conjunction with cognitive strategies and involves using adaptive sports and movement experiences to build in behavioral skills, which can be more accessible and meaningful for this population. The results provide recommendations for practice for the integration of certified therapeutic recreation specialists and adapted physical activity programs in the existing disability support services and mental health programs of educational institutions, including Minia University, with a focus on students’ well-being that goes beyond academic or medical solutions. Further research is needed to refine TPA protocols, use dismantling methods, objective outcome measures, and to conduct longitudinal follow-up to expand and clarify long-term outcomes and implementation of TPA in various disability and cultural contexts with the aim of achieving equitable mental health outcomes in the national and higher education systems.

## Data Availability

The datasets presented in this study can be found in online repositories. The names of the repository/repositories and accession number(s) can be found in the article/Supplementary material.
