# Supplementary material for: Therapeutic physical activity as a recreational intervention for anger control among students with physical disability: a behavioral model
Source: Front Behav Neurosci. 2026 Oct 16;20:1969746. doi: 10.3389/fnbeh.2026.1969746 (PMC13719462; doi:10.3389/fnbeh.2026.1969746)
Supplement: Supplementary file 1 [file Data_Sheet_1.PDF]

Appendix A.

PROMIS Anger Short Form 8a (Adaptation for University Students with Physical Disabilities)

No.	Item	Never	Rarely	Sometimes	Often	Always
**1**	I felt annoyed.					
**2**	I felt furious.					
**3**	I felt like yelling at someone.					
**4**	I had temper outbursts that I could not control.					
**5**	I felt irritable.					
**6**	I felt angry.					
**7**	I felt like I was losing my temper.					
**8**	I felt like exploding.					

Appendix B. Therapeutic Physical Activity (TPA) Intervention Protocol: Session-by-Session Outline

Week	Session	Phase & Duration	Core Activities & Behavioral Objectives	Materials & Adaptations
1	1	Warm-Up & Psychoeducation (15 min)<br>TPA Core (35 min)<br>Cool-Down (10 min)	Objective: Establish safety, build rapport, introduce anger-body connection.<br>• Ice-breaker adapted for mobility aids<br>• Group norms co-created with participants<br>• Seated rhythmic movement to music<br>• Guided body scan focusing on tension awareness	Bluetooth speaker, adaptive seating, visual schedule board
1	2	Warm-Up & Psychoeducation (15 min)<br>TPA Core (35 min)<br>Cool-Down (10 min)	Objective: Identify personal anger triggers through movement.<br>• Dynamic stretching with cognitive reframing prompts<br>• "Trigger Mapping" relay: move to stations representing different triggers<br>• Progressive muscle relaxation (seated/standing variants)	Trigger cards, resistance bands, relaxation audio
2	3	Warm-Up & Psychoeducation (15 min)<br>TPA Core (35 min)<br>Cool-Down (10 min)	Objective: Practice frustration tolerance under physical exertion.<br>• Cooperative relay races with intentional obstacles<br>• Assertive communication drills during task transitions<br>• Breathing regulation integrated into movement pauses	Cones, relay batons (adaptive grip), timer
2	4	Warm-Up & Psychoeducation (15 min)<br>TPA Core (35 min)<br>Cool-Down (10 min)	Objective: Develop physiological arousal reduction skills.<br>• Mindfulness-integrated balance exercises<br>• Wheelchair yoga flow targeting stress release<br>• Journaling prompt: "When my body feels calm, my mind feels..."	Yoga mats (wheelchair-accessible), journals, pens
3	5	Warm-Up & Psychoeducation (15 min)<br>TPA Core (35 min)<br>Cool-Down (10 min)	Objective: Build competence through mastery experiences.<br>• Skill-building circuit: progressively graded tasks<br>• Positive reinforcement scheduling for effort (not outcome)<br>• Reflection on "small wins" and self-efficacy	Adaptive sports equipment, reward tokens, reflection sheets
3	6	Warm-Up & Psychoeducation (15 min)<br>TPA Core (35 min)<br>Cool-Down (10 min)	Objective: Practice incompatible responses to anger cues.<br>• Simulated conflict scenarios during team games<br>• Replacement behavior rehearsal (e.g., deep breath → verbal request)<br>• Cool-down with guided imagery of safe spaces	Scenario cards, calming scent diffuser (optional)
4	7	Warm-Up & Psychoeducation (15 min)<br>TPA Core (35 min)<br>Cool-Down (10 min)	Objective: Enhance social connection and reduce isolation-related anger.<br>• Partner-based adaptive dance/movement<br>• Non-verbal communication exercises<br>• Group debrief on belonging and acceptance	Music playlist, partner matching system
4	8	Warm-Up & Psychoeducation (15 min)<br>TPA Core (35 min)<br>Cool-Down (10 min)	Objective: Integrate cognitive and behavioral skills in complex contexts.<br>• Multi-step obstacle course with decision points<br>• Stimulus control practice (identifying/modifying environmental triggers)<br>• Structured journaling linking session experiences to daily life	Obstacle course materials, trigger modification worksheets
5–8	9–16	Rotating Structure (Same 3-phase format)	Progressive Goals:<br>• Weeks 5–6: Advanced frustration tolerance + peer support roles<br>• Weeks 7–8: Relapse prevention planning + celebration of growth<br>• Each session builds on prior skills with increased complexity and autonomy	All previous materials + relapse prevention workbooks, certificates

Therapeutic Physical Activity (TPA) Protocol

Week 1: Foundation & Embodied Awareness (Sessions 1-2) *Objective:* Establish psychological safety, build therapeutic rapport, and introduce the connection between physical sensations and emotional states.

- **Session 1: Acquaintance & Building Trust in an Adaptive Space**
  - **Content:** Ice-breaking games specifically adapted for various mobility aids (e.g., wheelchair tag, seated ball toss); collaborative creation of group norms emphasizing respect and accessibility; guided body scan focusing on identifying areas of tension vs. relaxation while seated or using assistive devices.
  - **Techniques:** Positive Reinforcement, Socialization, Somatic Awareness.
  - **Executive Goal:** To create a safe, inclusive environment where participants feel physically comfortable and psychologically secure to express emotions.
- **Session 2: Mapping Anger Triggers Through Movement**
  - **Content:** Dynamic stretching integrated with cognitive prompts ("What makes your muscles tense when you're angry?"); "Trigger Mapping" relay where participants move to designated stations representing different triggers (e.g., unfairness, interruption); progressive muscle relaxation (seated/standing variants) to identify early physiological warning signs.
  - **Techniques:** Self-Monitoring, Psychoeducation, Adapted Movement.
  - **Executive Goal:** To develop embodied self-awareness of anger sources and recognize physical precursors before escalation.

Week 2: Cognitive Reframing & Physiological Regulation (Sessions 3-4) *Objective:* Learn to identify and restructure "hot thoughts" and acquire immediate tools for de-escalation through breath and movement.

- **Session 3: The Cognitive Connection: Thoughts, Body, and Anger**
  - **Content:** Identifying automatic negative thoughts during low-intensity rhythmic movement; role-playing scenarios while maintaining controlled breathing; reframing exercises paired with adaptive resistance band work to symbolize "letting go" of rigid thinking.
  - **Techniques:** Cognitive Restructuring, Modelling, Integrated Movement.
  - **Executive Goal:** To disrupt rumination cycles by linking cognitive reappraisal with proprioceptive feedback.
  -

- **Session 4: Immediate De-escalation: Breath and Balance**
  - **Content:** Practicing 4-7-8 breathing technique during balance challenges; mindfulness-integrated wheelchair yoga flow targeting stress release; journaling prompt: "When my body feels calm despite challenge, my mind feels..."
  - **Techniques:** Relaxation Training, Mindfulness, Adaptive Yoga.
  - **Executive Goal:** To anchor calming techniques in physical experience, making them accessible during moments of high arousal.

Week 3: Assertiveness & Frustration Tolerance (Sessions 5-6) *Objective:* Develop non-aggressive communication skills and practice tolerating frustration within structured physical tasks.

- **Session 5: Assertive Communication in Adaptive Contexts**
  - **Content:** Learning the "I feel... when... because..." model; practicing assertive statements during partner-based adaptive dance/movement drills; receiving positive reinforcement for clear, non-aggressive expression of needs related to accessibility or support.
  - **Techniques:** Role-Playing, Modelling, Behavioral Rehearsal.
  - **Executive Goal:** To express boundaries and needs clearly without aggression, reducing frustration from unmet needs.
- **Session 6: Practicing Incompatible Responses Under Exertion**
  - **Content:** Simulated conflict scenarios during cooperative relay races with intentional obstacles; rehearsing replacement behaviors (e.g., deep breath → verbal request) when frustrated by task difficulty; cool-down with guided imagery of safe spaces.
  - **Techniques:** Response Cost, Stimulus Control, Cooperative Play.
  - **Executive Goal:** To replace aggressive impulses with regulated responses even when physically challenged or frustrated.

Week 4: Emotional Expression & Cooperative Mastery (Sessions 7-8) *Objective:* Channel intense emotions safely and build competence through collaborative achievement.

- **Session 7: Creative Expression: Art as Emotional Release**
  - **Content:** Using adaptive art tools (e.g., mouth sticks, head pointers, modified grips) to draw/sculpt "what anger looks like"; voluntary group sharing focused on validation rather than judgment; transitioning from creative expression to gentle movement.

    - **Techniques:** Emotional Discharge, Expressive Arts Therapy, Validation.
    - **Executive Goal:** To provide a non-verbal, safe outlet for processing complex emotions related to disability and exclusion.
- **Session 8: Cooperative Tug-of-War: Strategy Over Strength**
    - **Content:** Modified tug-of-war emphasizing teamwork, communication, and strategy over sheer physical power; debriefing on how collaboration reduced individual frustration; reinforcing effort and mutual support.
    - **Techniques:** Socialization, Positive Reinforcement, Team Building.
    - **Executive Goal:** To redirect physical energy into cooperative problem-solving and experience mastery through collective effort.

Week 5: Problem-Solving & Sportsmanship (Sessions 9-10) *Objective:* Apply structured conflict resolution methods and practice emotional regulation in competitive sports settings.

- **Session 9: Structured Conflict Resolution**
    - **Content:** Learning step-by-step problem-solving (define, brainstorm, choose) through adapted team games; role-playing disagreements about game rules or fairness; practicing calm negotiation during active play.
    - **Techniques:** Cognitive-Behavioral Problem Solving, Social Skills Training.
    - **Executive Goal:** To internalize a practical methodology for resolving interpersonal conflicts calmly and constructively.
- **Session 10: Basketball for Teamwork & Self-Control**
    - **Content:** Adaptive basketball drills focused on passing, supporting teammates, and accepting outcomes; short scrimmage with mandatory "pause-and-breathe" breaks after fouls or missed shots; modeling sportsmanship by facilitators.
    - **Techniques:** Reinforcement, Modelling, Applied Sport Psychology.
    - **Executive Goal:** To practice impulse control and emotional regulation under the natural pressure of competition.

Week 6: Advanced Coping & Focus (Sessions 11-12) *Objective:* Master strategic withdrawal and enhance attentional control through agility-based activities.

- **Session 11: Smart Withdrawal & Time-Out Strategy**
    - **Content:** Practicing recognizing personal escalation cues during physical activity; rehearsing effective communication of need for break ("I need a moment to reset"); using designated "calm zones" within the activity space without penalty.

- **Techniques:** Self-Regulation, Response Prevention, Environmental Modification.
- **Executive Goal:** To normalize voluntary time-out as a proactive coping skill, not avoidance or failure.

- **Session 12: Agility & Focus: Jumping Inside Hoops**
  - **Content:** Adaptive agility games using hula hoops (sequential jumping, relay races) emphasizing rule-following, turn-taking, and maintaining composure despite errors; reinforcing focus and persistence over speed.
  - **Techniques:** Attention Training, Behavioral Shaping, Adaptive PE.
  - **Executive Goal:** To improve sustained attention and impulse control through structured, rule-bound physical challenges.

Week 7: Integration & High-Arousal Application (Sessions 13-14) *Objective:* Synthesize all learned skills in complex, emotionally evocative scenarios.

- **Session 13: Comprehensive Skill Integration Role-Play**
  - **Content:** Complex scenarios combining triggers, negative thoughts, assertive communication, and time-out use during adaptive team challenges; peer feedback on skill application; facilitator modeling of integrated responses.
  - **Techniques:** Comprehensive Behavioral Rehearsal, Peer Coaching, Generalization Training.
  - **Executive Goal:** To demonstrate fluent use of the full anger management toolkit in realistic, multi-demand situations.
- **Session 14: Football for Sportsmanship & Emotional Acceptance**
  - **Content:** Adaptive football match with explicit focus on respecting opponents, accepting referee decisions, and managing disappointment; post-game debrief linking emotional experiences to specific strategies used; celebrating effort over outcome.
  - **Techniques:** Applied Sport Psychology, Positive Reinforcement, Reflective Practice.
  - **Executive Goal:** To test and consolidate emotional regulation skills in a high-arousal, socially complex team environment.

Week 8: Consolidation, Relapse Prevention & Celebration (Sessions 15-16) *Objective:* Solidify learning, plan for maintenance, and end with positive reinforcement and closure.

- **Session 15: Review & Personalized Relapse Prevention Plan**

- **Content:** Group discussion identifying most useful skills and future high-risk situations; creating individualized "Anger Management Action Plans" incorporating adaptive strategies; peer support commitments for ongoing accountability.
- **Techniques:** Relapse Prevention Planning, Social Support Activation, Self-Efficacy Enhancement.
- **Executive Goal:** To empower participants to independently apply and maintain skills beyond the program's conclusion.

- **Session 16: Closure, Celebration & Affirmation**
  - **Content:** Sharing positive feedback and growth reflections; awarding certificates of participation; final inclusive recreational activity/snack; facilitated closing ritual affirming each participant's progress and worth.
  - **Techniques:** Positive Reinforcement, Ritual Closure, Strengths-Based Affirmation.
  - **Executive Goal:** To reinforce achievement, foster positive associations with the intervention, and provide dignified, celebratory closure.
